# The effects of intermittent hypoxic training on the aerobic capacity of exercisers: a systemic review and meta-analysis

**DOI:** 10.1186/s13102-023-00784-3

**Published:** 2023-12-19

**Authors:** Zhihao Huang, Shulin Yang, Chunyang Li, Xingchao Xie, Yongming Wang

**Affiliations:** 1 School of Big Data and Fundamental Sciences, Shandong Institute of Petroleum and Chemical Technology, Dongying, China; 2 School of Sports Sciences, Nanjing Normal University, Nanjing, China

**Keywords:** Intermittent hypoxic training, Exercisers, Aerobic capacity, Maximal oxygen uptake, Hemoglobin, Meta-analysis

## Abstract

**Objective:**

To systematically review the effects of intermittent hypoxic training on the aerobic capacity of exercisers.

**Methods:**

PubMed, Embase, The Cochrane Library, and Web of Science databases were electronically searched to collect studies on the effects of intermittent hypoxic training on the aerobic capacity of exercisers from January 1, 2000, to January 12, 2023. Two reviewers independently screened the literature, extracted data, and assessed the risk of bias of the included studies. Then, meta-analysis was performed by using Stata SE 16.0 software.

**Results:**

A total of 19 articles from 27 studies were included. The results of the meta-analysis showed that compared with the control group, the intermittent hypoxic training group had significantly increased maximal oxygen uptake [weighted mean difference = 3.20 (95%CI: 1.33 ~ 5.08)] and hemoglobin [weighted mean difference = 0.25 (95%CI: 0.04 ~ 0.45)].

**Conclusion:**

Intermittent hypoxic training can significantly improve the aerobic capacity of exercisers. Due to the limited quantity and quality of the included studies, more high-quality studies are needed to verify the above conclusion.

**Supplementary Information:**

The online version contains supplementary material available at 10.1186/s13102-023-00784-3.

## Introduction

Since the 1968 Mexico City Olympic Games, held at high altitude, the efficacy of altitude or hypoxic training for enhancing aerobic exercise performance has garnered significant interest among athletes, coaches, and researchers [1]. Several strategies of altitude training, like “live high + train high”, “live high + train low” and “live low + train high” have been proposed [2–4]. High altitudes lead to reduced partial pressure of oxygen due to lower atmospheric pressure, resulting in decreased alveolar partial pressure of oxygen, arterial oxygen saturation, and arteriovenous oxygen difference, thereby diminishing oxygen delivery and utilization capacity [5–7]. As a result, performing exercises at the same intensity feels harder in hypoxia, compared with normoxia, with increased ventilation, heat rate, cardiac output, lactate concentration, and oxygen consumption during submaximal exercise [8, 9]. However, altitude training is constrained by factors such as high travel costs, specific geographical locations, and prolonged recovery periods for bodily functions. In-depth studies of altitude training have yielded various training modes that simulate hypoxic environments. Intermittent hypoxic training (IHT) is one such approach, entailing discontinuous exposure to normobaric or hypobaric hypoxia to replicate key aspects of altitude acclimatization and ultimately improve sea-level athletic performance. IHT generally encompasses two strategies: inducing hypoxia at rest to primarily stimulate altitude acclimatization or inducing hypoxia during exercise to primarily augment the training stimulus [10]. IHT’s role in hypoxic training modalities has been increasingly emphasized as a means to not only address the limitations of traditional altitude training but also enhance exercisers’ performance capabilities. This study focuses on maximal oxygen uptake (VO_2__max_) and hemoglobin(HB), two factors closely related to aerobic capacity and subject to controversial research findings. The aim of this systematic review was to synthesize and analyze the existing literature on the effects of interventions on aerobic metabolic capacity, with a specific focus on identifying the impact of IHT on VO_2__max_ and HB concentration in exercisers.

## Materials and methods

In this study, we followed the Preferred Reporting Items for Systematic Reviews and Meta-Analyses 2020 guidelines [11] and registered our review with the International Prospective Register of Systematic Reviews (registration number: CRD42023407552).

### Search strategy

A literature search was performed by two independent reviewers. PubMed, Embase, The Cochrane Library, and Web of Science were searched from January 1, 2000, to January 12, 2023. To minimize the missing literature, references listed in the included studies were also traced to supplement relevant data.

### Eligibility criteria

Using the PICOS approach, the inclusion criteria were specified as follows: Participants in the studies were exercisers. The intervention of interest revolved around IHT design schemes. In comparison, the control group was specified as the normobaric group. Primary outcome indices under scrutiny included measures such as VO_2__max_ and HB. Pertaining to the study design, all considered study designs were randomized controlled trials, with no particular emphasis on whether blinding was employed or if allocation concealment was enacted.

The exclusion criteria were as follows: duplicate articles; articles with inconsistent research contents; review articles; conference abstracts; animal studies; case reports; study protocols; and non-English and non-Chinese articles.

### Literature screening and data extraction

Two independent reviewers conducted the literature screening and data extraction, cross-checking their findings. In cases of disagreement, a third party was consulted to provide adjudication. Following the exclusion of irrelevant literature, the full texts of the remaining potentially relevant articles were thoroughly examined to determine their inclusion in the final analysis. Data extraction encompassed the basic characteristics of the included studies, such as author, publication year, country, and outcomes.

### Risk of bias assessment of the included studies

The risk of bias (ROB) assessment was conducted independently by two reviewers using the Cochrane Collaboration’s tool [12]. Critical assessments were made separately for each type of bias, including selection bias, performance bias, detection bias, attrition bias, reporting bias, and other biases. In cases of disagreement, a third party was consulted to provide adjudication.

### Statistical analysis

Review Manager 5.3 software (The Cochrane Collaboration, UK) was utilized to assess the ROB of the included studies. Stata SE 16.0 software (Computer Resource Center, USA) was employed for meta-analysis, using weighted mean difference (WMD) as the statistic for effect analysis and providing a 95% confidence interval. Heterogeneity of meta-analysis results was tested using I². An I² value greater than 50% indicated significant heterogeneity among studies [13, 14], necessitating the use of a random-effects model if significant heterogeneity could not be resolved through meta-regression or subgroup analysis. Otherwise, a fixed-effects model was employed. Sensitivity analysis was conducted by excluding included studies one at a time [15]. Funnel plots were used to evaluate the presence of publication bias [16]: a P-value greater than 0.05 in Begg’s test indicated no publication bias, whereas a P-value less than or equal to 0.05 indicated publication bias.

## Results

### Literature search

**Fig. 1:**
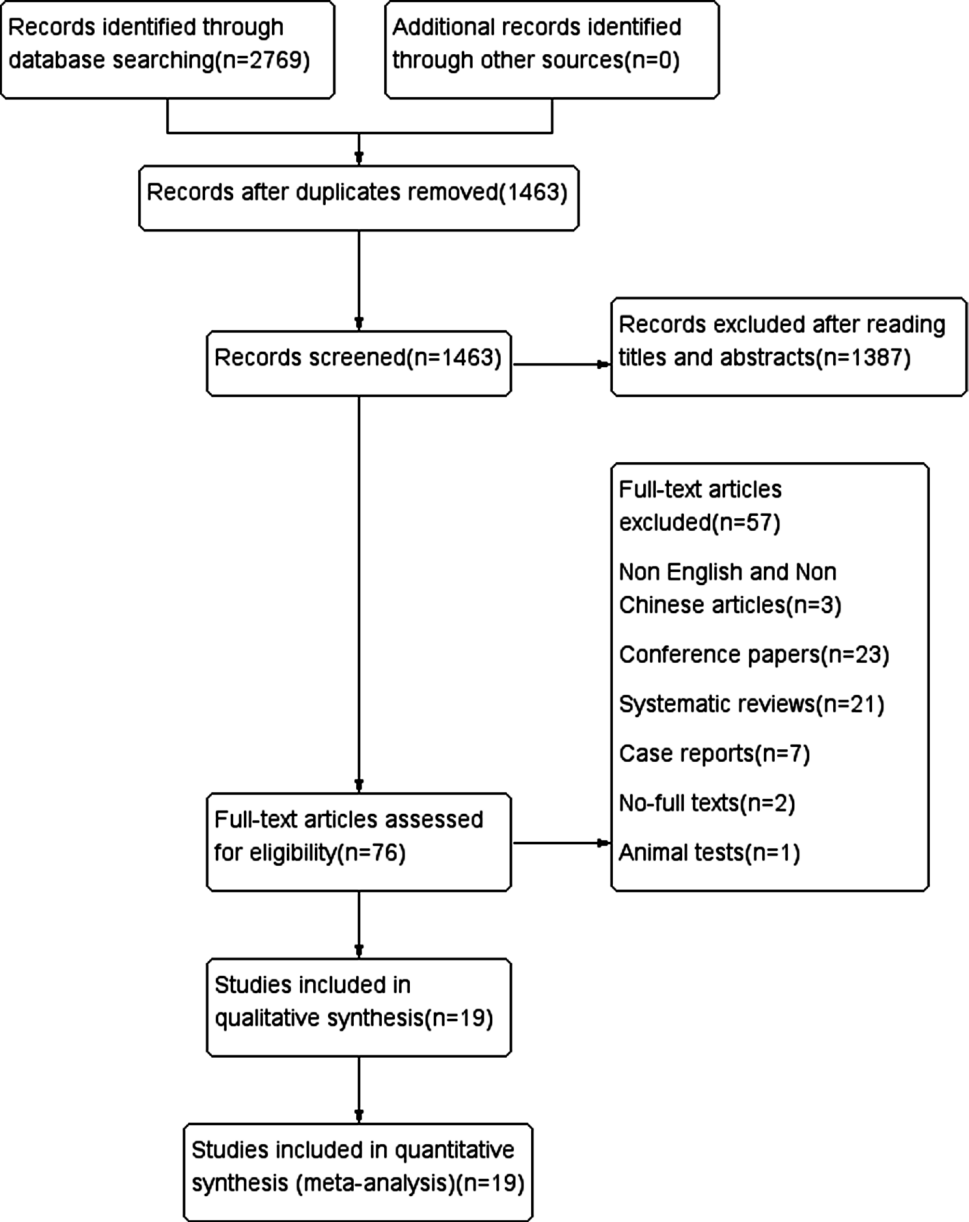
Flow diagram of the literature search and selection process

Pursuant to the search strategy, the initial search identified 2,769 articles. Detailed information can be found in Supplemental Method 1. After a step-by-step screening process, a total of 19 articles were ultimately included [17–35]. The literature screening process and results are illustrated in Fig. 1.

### Detailed information and ROB results

**Fig. 2:**
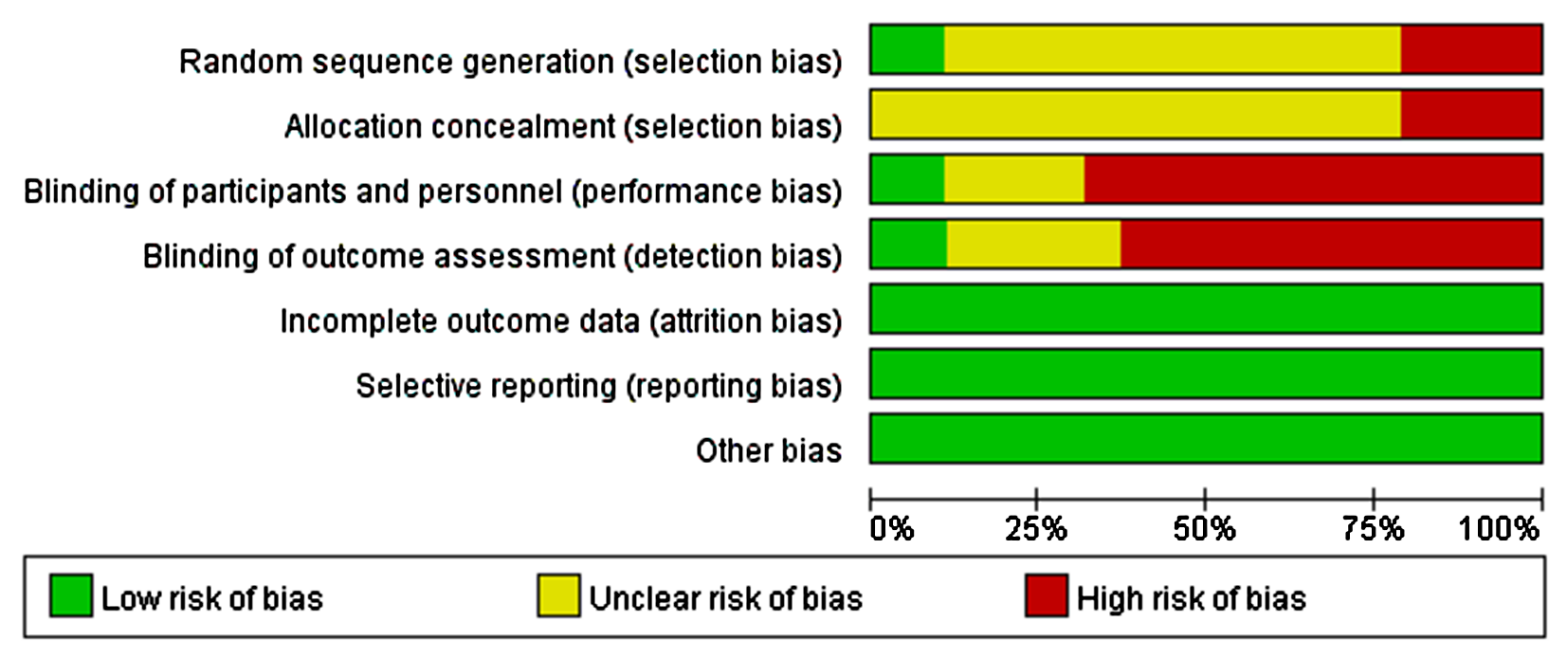
Risk of bias and applicability concerns graph

**Fig. 3:**
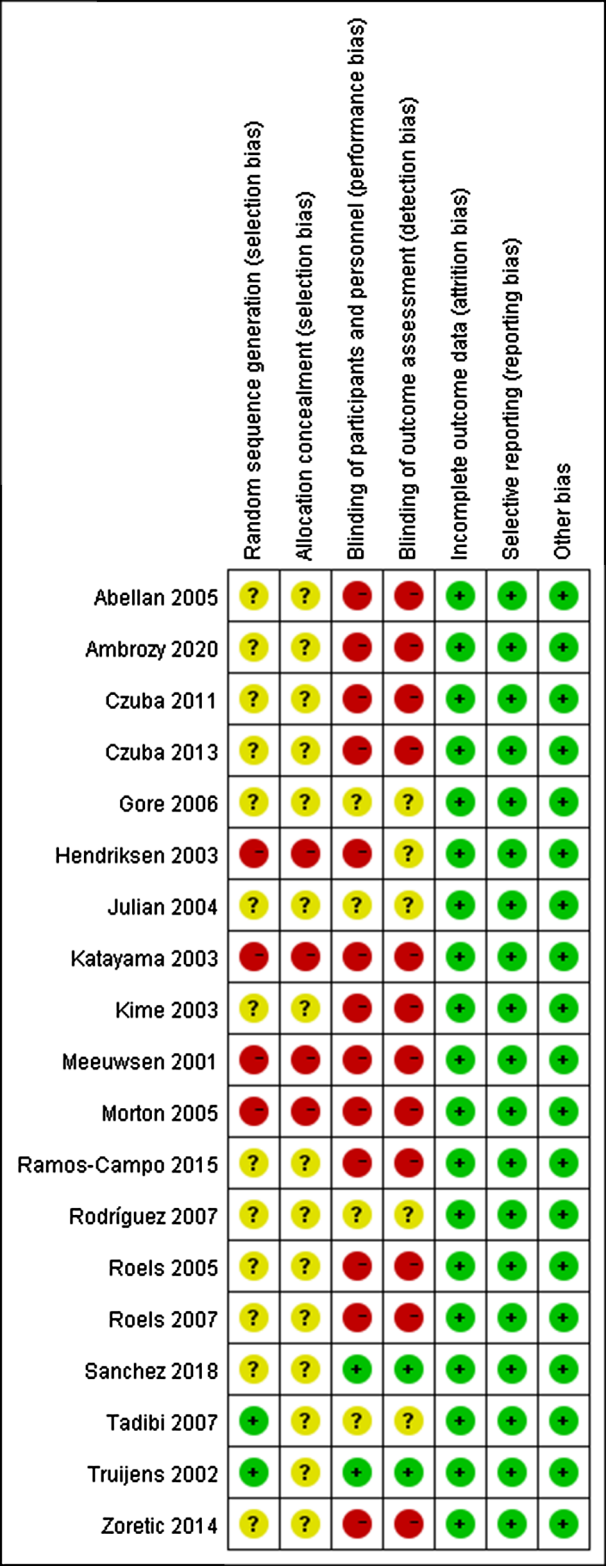
Risk of bias and applicability concerns summary

Comprehensive information for the included studies is presented in Table 1. The ROB results for the included studies can be found in Figs. 2 and 3.

**Table 1:** Characteristics of the included studies

Study name	Country	Methods of simulating altitude	Simulated altitude	Intervention method	Intervention period	Time of IHT delivered	Sport event	Age	Sample size(male/female)
Abellan 2005 [17]	Spain	Not available	Simulated altitude of 4,000 ~ 5,500 m	3 h/day, 5 days/week	4 weeks	Rest	Triathlon	Not available	16(16/0)
Ambrozy 2020 [18]	Poland	Altering FiO_2_ to 12.9%	Simulated altitude of 4,000 m	1 h/day, 5 days/week	6 weeks	Exercise	Boxing	23.9 ± 2 0.97	30(30/0)
Czuba 2011 [19]	Poland	Altering O_2_ of environment to 15.2%	Simulated altitude of 2,500 ~ 2,600 m	1.5 ~ 2 h/day, 3 days/week	3 weeks	Exercise	Cycling	22.75 ± 3.14	20(20/0)
Czuba 2013 [20]	Poland	Altering FiO_2_ to 15.2%	Simulated altitude of 2,500 m	1.5 ~ 2 h/day, 3 days/week	3 weeks	Exercise	Basketball	22.0 ± 1.94	20(20/0)
Gore 2006 [21]	Australia	US Air Force standard atmospheric tables	Simulated altitude of 4,000 ~ 5,500 m	3 h/day, 5 days/week	4 weeks	Rest	Running and swimming	22.8 ± 8.04	27(16/11)
Hendriksen 2003 [22]	Netherlands	Not available	Simulated altitude of 2,500 m	2 h/day, 3 days/week	9 days	Exercise	Triathlon	Not available	24(24/0)
Julian 2004 [23]	USA	Altering FiO_2_ to a range of 10.0–12.0%	Not available	70 min/day, 5 days/week	4 weeks	Rest	Running	25.1 ± 3.23	17(14/3)
Katayama 2003 [24]	Japan	Lowing barometric pressure to 432 torr	Simulated altitude of 4,500 m	1.5 h/day, 3 days/week	3 weeks	Rest	Running	21.6 ± 2.93	12(12/0)
Kime 2003 [25]	USA	Altering FiO_2_ to 15.0%	Simulated altitude of 2,500 m	2 h/day, 3 days/week	3 weeks	Exercise	Cycling	17.0 ± 1.00	8(7/1)
Meeuwsen 2001 [26]	Netherlands	Not available	Simulated altitude of 2,500 m	2 h/day	10 days	Exercise	Triathlon	29.0(21 ~ 39)	14(14/0)
Morton 2005 [27]	UK	Altering FiO_2_ to 15.0%	Simulated altitude of 2,750 m	30 min/day, 3 days/week	4 weeks	Exercise	Team sports	20.5 ± 0.80	16(16/0)
Ramos-Campo 2015 [28]	Spain	Altering FiO_2_ to a range of 14.5–15.0%	Not available	1 h/day, 2 days/week	7 weeks	Rest	Triathlon	27.7 ± 6.77	18(18/0)
Rodríguez 2007 [29]	USA	Altering FiCO_2_ to less than 0.2%	Simulated altitude of 4,000 ~ 5,500 m	3 h/day, 5 days/week	4 weeks	Rest	Running and swimming	22.5 ± 8.10	23(12/11)
Roels 2005 [30]	France	Altering PiO_2_ of 100 mm Hg	Simulated altitude of 3,000 m	114.9 min/week	3 weeks	Exercise	Cycling and triathlon	26.6 ± 6.25	19(NA)
Roels 2007 [31]	France	Altering PiO_2_ of 100 mm Hg	Simulated altitude of 3,000 m	1 ~ 1.5 h/day, 5 days/week	3 weeks	Exercise	Cycling and triathlon	24.3 ± 0.35	18(NA)
Sanchez 2018 [32]	France	Altering FiO_2_ to a range of 10.6–11.4%	Simulated altitude of 5,000 ~ 5,500 m	1 h/day, 3 days/week	6 weeks	Exercise	Running	28.5 ± 9.21	15(15/0)
Tadibi 2007 [33]	Germany	Altering O_2_ of environment to a range of 10.0–11.0%	Simulated altitude of 5,200 ~ 5,900 m	1 h/day, 7 days/week	15 days	Exercise	Running	27.7 ± 4.16	20(20/0)
Truijens 2002 [34]	USA	Altering FiO_2_ to 15.2%	Simulated altitude of 2,500 m	20 min/day, 7 days/week	5 weeks	Exercise	Swimming	28.8 ± 10.04	16(6/10)
Zoretic 2014 [35]	Croatia	Altering EtCO_2_ of 45 mm Hg	Not available	30 ~ 45 min/day, 3 days/week	8 weeks	Exercise	Swimming	15 ~ 25	16(16/0)

### Meta-analysis of the effect of IHT on VO_2__max_

**Fig. 4:**
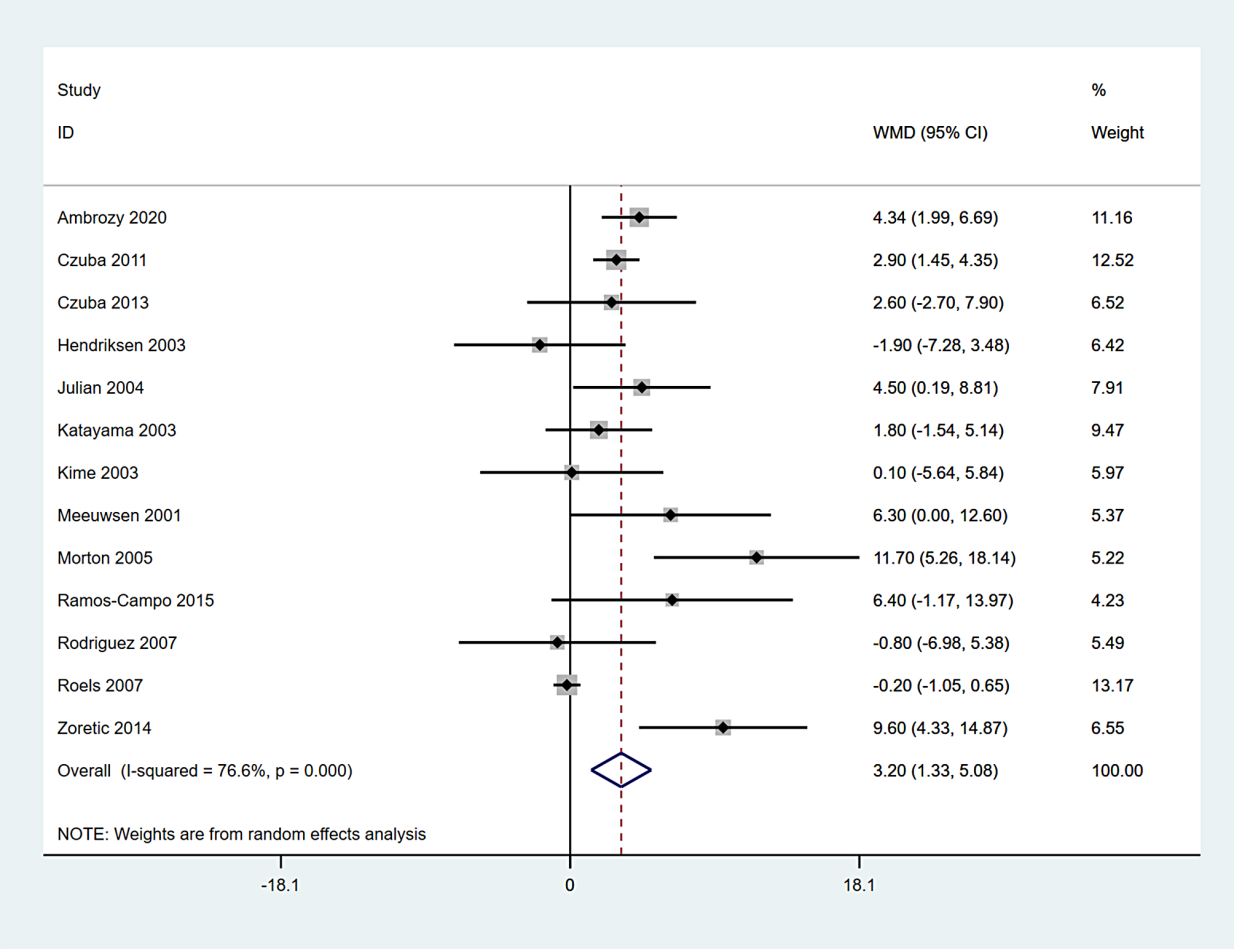
Forest plot for pooled effect size of VO_2_ _max_ outcome (ml/kg/min)

**Fig. 5:**
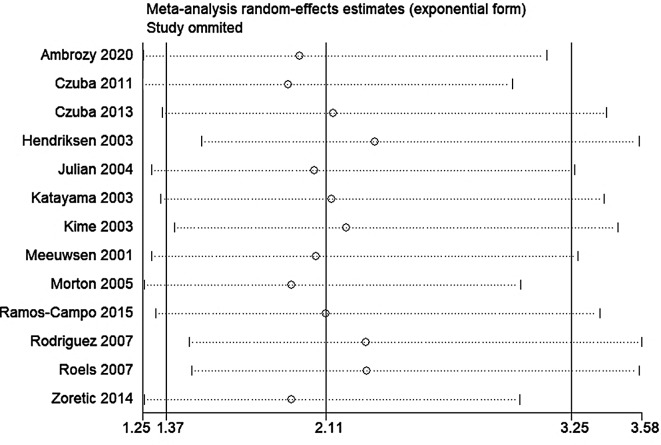
Sensitivity analysis of VO_2 max_ outcome

**Fig. 6:**
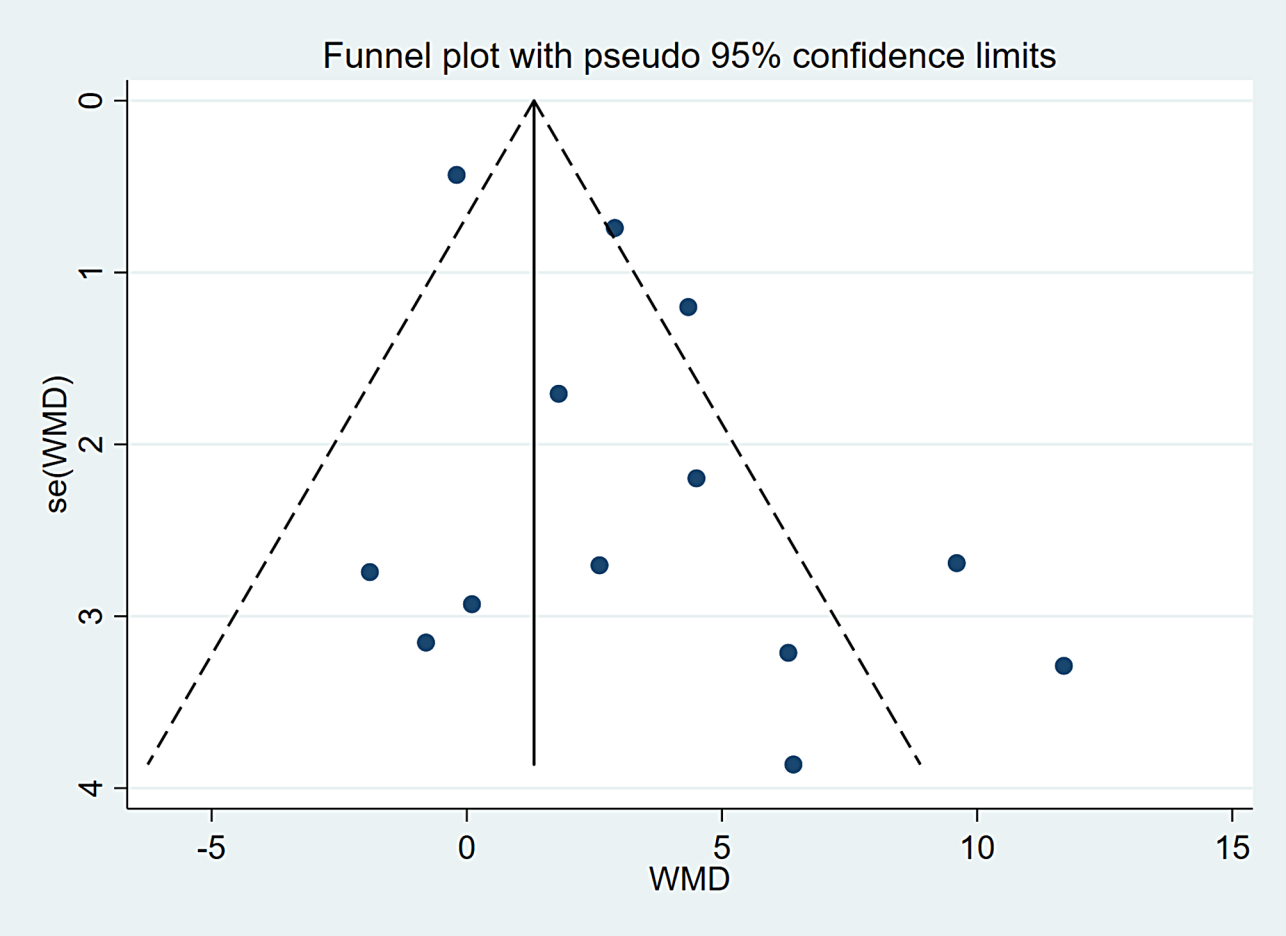
Funnel plot of VO_2 max_ outcome

A total of 13 studies encompassing 225 participants were included (Table 2). Heterogeneity test results revealed substantial heterogeneity among the studies (I² = 76.6%, P = 0.000) (Fig. 4). The cause of heterogeneity was not identified through meta-regression or subgroup analysis; thus, a random-effects model was employed to pool effect size. The effect size WMD was 3.20 ml/kg/min (95%CI: 1.33 ~ 5.08) (Fig. 4). After sequentially excluding individual studies, the remaining studies were reanalyzed in a pooled manner. The results indicated that each excluded study had a minor impact on the magnitude of the effect size, suggesting that the meta-analysis results were stable and reliable (Fig. 5). A funnel plot was constructed using se(WMD) on the ordinate axis and WMD on the abscissa (Fig. 6). The findings demonstrated a Begg’s test P-value of 0.760, indicating no publication bias.

**Table 2:** The summary statistics of intermittent hypoxic training on maximal oxygen uptake (ml/kg/min)

Study	Methods to determine VO_2_ _max_	n_Exp	Mean_Exp	SD_Exp	n_Cont	Mean_Cont	SD_Cont
Ambrozy 2020 [18]	Progressive test	15	57.07	3.13	15	52.73	3.44
Czuba 2011 [19]	Progressive test	10	70.50	1.50	10	67.60	1.80
Czuba 2013 [20]	Progressive test	6	56.70	4.10	6	54.10	5.20
Hendriksen 2003 [22]	Progressive test	12	65.70	8.20	12	67.60	4.80
Julian 2004 [23]	Progressive test	7	76.10	4.40	7	71.60	3.80
Katayama 2003 [24]	Progressive test	6	69.50	3.10	6	67.70	2.80
Kime 2003 [25]	Progressive test	4	59.80	2.70	4	59.70	5.20
Meeuwsen 2001 [26]	Progressive test	7	72.20	5.10	7	65.90	6.80
Morton 2005 [27]	Progressive test	8	56.80	7.50	8	45.10	5.50
Ramos-Campo 2015 [28]	Progressive test	9	65.50	4.90	9	59.10	10.5
Rodríguez 2007 [29]	Progressive test	11	57.00	8.80	12	57.80	5.90
Roels 2007 [31]	Progressive test	10	48.60	0.64	8	48.80	1.08
Zoretic 2014 [35]	Indirect test	8	70.40	5.26	8	60.80	5.50

### Meta-analysis of the effect of IHT on HB

**Fig. 7:**
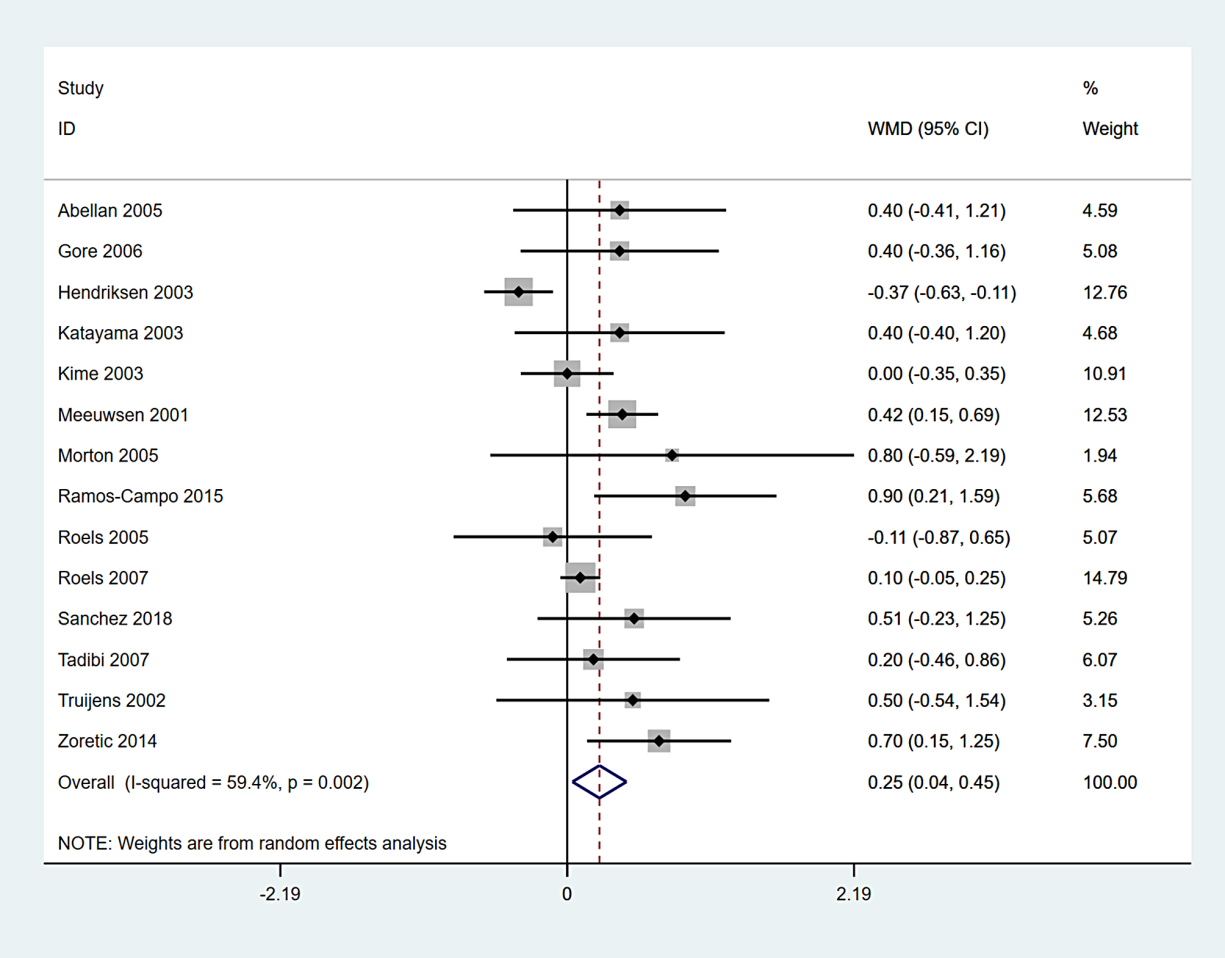
Forest plot for pooled effect size of HB outcome (g/L)

**Fig. 8:**
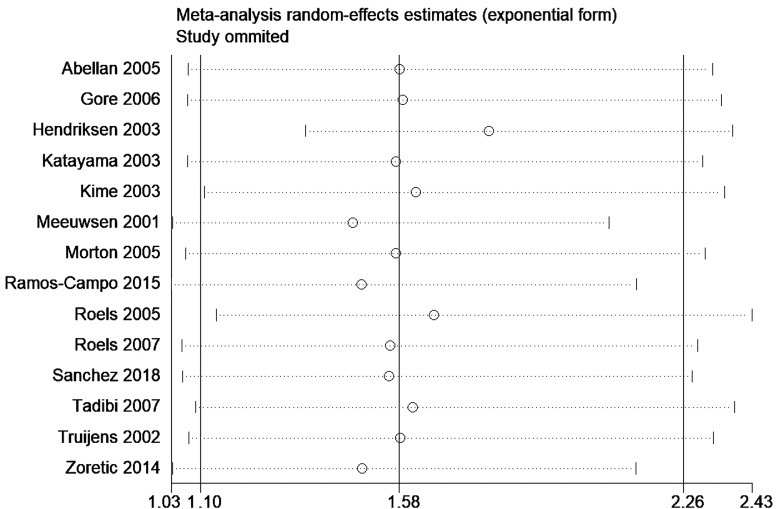
Sensitivity analysis of HB outcome

**Fig. 9:**
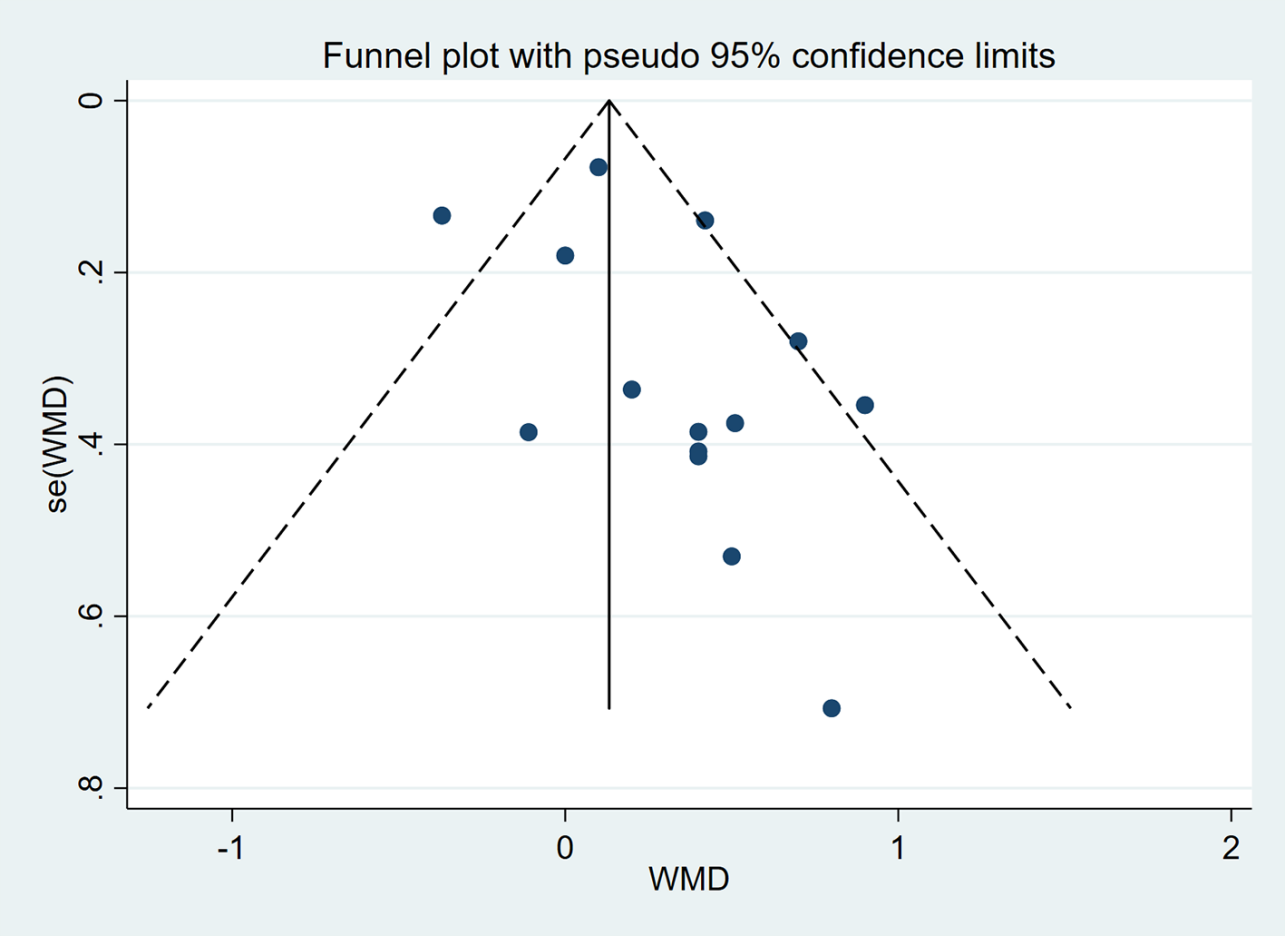
Funnel plot of HB outcome

A total of 14 studies encompassing 234 participants were included (Table 3). Heterogeneity test results revealed substantial heterogeneity among the studies (I^2^ = 59.4%, P = 0.002) (Fig. 7). The cause of heterogeneity was not identified through meta-regression or subgroup analysis; thus, a random-effects model was employed to pool effect size. The effect size WMD was 0.25 g/L (95%CI: 0.04 ~ 0.45) (Fig. 7). After sequentially excluding individual studies, the remaining studies were reanalyzed in a pooled manner. The results indicated that each excluded study had a minor impact on the magnitude of the effect size, suggesting that the meta-analysis results were stable and reliable (Fig. 8). A funnel plot was constructed using se(WMD) on the ordinate axis and WMD on the abscissa (Fig. 9). The findings demonstrated a Begg’s test P-value of 0.661, indicating no publication bias.

**Table 3:** The summary statistics of intermittent hypoxic training on hemoglobin (g/L)

Study	n_Exp	Mean_Exp	SD_Exp	n_Cont	Mean_Cont	SD_Cont
Abellan 2005 [17]	8	14.30	1.10	8	13.90	0.40
Gore 2006 [21]	10	14.60	0.90	12	14.20	0.90
Hendriksen 2003 [22]	12	9.21	0.25	12	9.58	0.39
Katayama 2003 [24]	6	15.00	0.60	6	14.60	0.80
Kime 2003 [25]	4	14.40	0.20	4	14.40	0.30
Meeuwsen 2001 [26]	7	9.65	0.24	7	9.23	0.28
Morton 2005 [27]	8	15.30	1.60	8	14.50	1.20
Ramos-Campo 2015 [28]	9	15.40	0.80	9	14.50	0.70
Roels 2005 [30]	11	14.75	1.01	8	14.86	0.67
Roels 2007 [31]	10	14.80	0.10	8	14.70	0.20
Sanchez 2018 [32]	9	16.08	0.73	6	15.57	0.70
Tadibi 2007 [33]	10	15.00	0.70	10	14.80	0.80
Truijens 2002 [34]	8	14.70	1.20	8	14.20	0.90
Zoretic 2014 [35]	8	15.20	0.56	8	14.50	0.56

## Discussion

Hypoxic training is a method that simulates high-altitude hypoxic environments using hypoxic instruments in plain conditions, providing appropriate hypoxic stimulation to exercisers to enhance their aerobic metabolism and hypoxia tolerance. Considering the benefits of hypoxic training, including improved aerobic metabolic efficiency and augmented tolerance to hypoxia, current investigations into this training modality have captured substantial interest from the global sports science research community. [36, 37]. The primary objective of this study was to examine the impact of IHT on exercisers’ aerobic capacity by assessing alterations in VO_2__max_ and HB. VO_2__max_ is an essential part of health and physical fitness, and refers to the highest rate of oxygen consumption an individual can attain during exhaustive exercise [38]. VO_2__max_ directly reflects the working capacity of the cardiovascular system and the body’s aerobic endurance. The higher the VO_2__max_, the stronger the aerobic capacity [39].HB, the oxygen carrying protein, ferries nearly all bodily oxygen from the lungs to cells and tissues in need [40]. The concentration and quality of HB can affect the ability to transport oxygen, which in turn influences aerobic exercise performance. Higher levels of HB generally mean better oxygen transport and higher aerobic endurance [41]. Theoretically, IHT enhances aerobic capacity and can improve athletic performance at sea level.

The findings of this study revealed that IHT had a significant impact on elevating exercisers’ VO_2__max_ (P < 0.05). Berezovskiĭ demonstrated that IHT augmented lung reserve and enhanced respiratory muscle strength in participants [42]. During IHT, the body continually receives hypoxic stimuli, which undoubtedly exercises the respiratory muscles and fosters adaptation, ultimately enhancing respiratory function. IHT involves the inhalation of gas with low oxygen partial pressure, leading to reduced arterial oxygen saturation, stimulation of carotid and aortic body chemoreceptors, and consequently, increased cardiovascular pulse, elevated blood pressure, and intensified respiratory effort. Following IHT, participants show improved lung and respiratory system function, which may contribute to enhanced aerobic performance. However, it’s worth noting that while lung function is associated with the absorption capacity of oxygen, it is not directly related to oxygen transport capacity, which is primarily influenced by levels of HB.

This study demonstrated that IHT induced significant alterations in exercisers’ HB levels (P < 0.05). HB is a widely recognized iron-containing protein in blood that is crucial for oxygen transport in mammals [43, 44]. HB facilitates oxygen transfer to muscle cells, assisting exercisers in sustaining their physical strength. Concurrently, HB plays a vital role in maintaining the acid-base balance of blood [45], thereby enhancing exercisers’ muscle tolerance to hypoxia. IHT significantly boosts exercisers’ HB levels by enhancing erythropoietin secretion from the kidneys, causing plasma volume reduction due to osmosis, increasing muscle efficiency in oxygen utilization, and maintaining acid-base balance in the blood, thereby improving oxygen transport capacity and hypoxia tolerance.

Upon reviewing the study designs of each article and combining them with the results of this meta-analysis, the sources of heterogeneity can be elucidated as follows. First, participant demographics varied: some studies focused on IHT for adolescent exercisers, while others targeted adult exercisers, some studies focused on IHT for individual sports, while others targeted team sports. Moreover, the performance level of the participants also influenced outcomes. For instance, when aerobic endurance reaches a high level, such as in elite endurance exercisers, VO_2__max_ becomes less effective at accurately reflecting individual aerobic endurance [46]. Second, training intensity contributed to heterogeneity: some studies employed low-intensity training, while others used medium- to high-intensity hypoxia training, yielding differing IHT effects for various exercisers. Last, the timing of experimental testing differed, although the pretest timing was consistent across the included studies, posttest timings varied: in some studies, testing occurred immediately following IHT; in others, it took place after a one-week interval or longer. The timing of these tests is partly responsible for heterogeneity. These factors could not be identified through meta-regression or subgroup analyses.

The study has several limitations to consider. Firstly, the exclusion of unpublished literature and the inability to fully collect some articles may affect the comprehensiveness of our findings. Secondly, by focusing only on articles written in English, we may have overlooked valuable insights from non-English sources. Thirdly, the underrepresentation of female exercisers can introduce bias into our results. Finally, concerns regarding risk of bias arise from the lack of details on random sequence generation, allocation concealment, and the use of blinding methods in some of the literature, potentially affecting the overall credibility of the study.

## Conclusion

IHT has shown significant positive effects on improving exercisers’ VO_2__max_ and HB concentration. These findings preliminarily support the use of IHT as a beneficial method for enhancing aerobic capacity. However, due to the limitations in the quality of the studies, these conclusions should be approached with caution and need to be verified by further high-quality research. Additionally, future research should strive to uncover more layers of IHT’s potential effects and explore its role in optimizing performance, to fully understand and utilize this training modality. For this purpose, accurately determining the specific impacts of IHT on exercisers with different levels of physical fitness, sports backgrounds, and genders will be an important direction of study. At the same time, how to optimize IHT protocols to achieve the maximum benefit of individualized training is a question worth exploring in depth within the field of sports science.

### Electronic supplementary material

Below is the link to the electronic supplementary material.

Supplementary Material 1

## Data Availability

All data generated or analyzed during this study are included in this published article and its Supplemental Digital Content. The datasets generated during and/or analyzed during the current study are publicly available.

## Abbreviations

IHT: Intermittent hypoxic training
VO_2__max_: Maximal oxygen uptake
HB: Hemoglobin
ROB: risk of bias
WMD: Weighted mean difference
