# Supplementary material for: The effects of intermittent hypoxic training on the aerobic capacity of exercisers: a systemic review and meta-analysis
Source: BMC Sports Sci Med Rehabil. 2023 Dec 19;15:174. doi: 10.1186/s13102-023-00784-3 (PMC10731756; doi:10.1186/s13102-023-00784-3)
Supplement: Supplementary file 1 — Supplementary Material 1 [file 13102_2023_784_MOESM1_ESM.doc]

**Supplemental Method 1** Electronic search strategies

**PubMed**

#1 ((intermittent hypoxic[Title/Abstract]) OR (interval training[Title/Abstract])) OR (intermittent hypoxia[Title/Abstract])

#2 ((((((((aerobic capacity[Title/Abstract]) OR (aerobic endurance[Title/Abstract])) OR (aerobic stamina[Title/Abstract])) OR (cardio[Title/Abstract])) OR (maximal oxygen consumption[Title/Abstract])) OR (VO_2max_[Title/Abstract])) OR (hemoglobin[Title/Abstract])) OR (haemoglobin[Title/Abstract])) OR (Hb[Title/Abstract])

#3 randomized controlled trial[Publication Type] OR randomized[Title/Abstract] OR placebo[Title/Abstract]

#4 #1 AND #2 AND #3

Note: We searched 410 articles while using PubMed as the filter.

**Embase**

#1 'intermittent hypoxic':ab,ti OR 'interval training':ab,ti OR 'intermittent hypoxia':ab,ti

#2 'aerobic capacity':ab,ti OR 'aerobic endurance':ab,ti OR 'aerobic stamina':ab,ti 0R 'cardio':ab,ti 0R 'maximal oxygen consumption':ab,ti 0R 'VO_2max_':ab,ti 0R 'hemoglobin':ab,ti 0R 'haemoglobin':ab,ti 0R 'Hb':ab,ti

#3 'randomized controlled trial':ab,ti OR 'randomized':ab,ti OR 'placebo':ab,ti

#4 #1 and #2 and #3

Note: We searched 276 articles while using RefMan-(RIS) as the filter.

**Cochrane Library**

#1 (intermittent hypoxic):ab,ti,kw OR (interval training):ab,ti,kw OR (intermittent hypoxia):ab,ti,kw

#2 (aerobic capacity):ab,ti,kw OR (aerobic endurance):ab,ti,kw OR (aerobic stamina):ab,ti,kw OR (cardio):ab,ti,kw 0R (maximal oxygen consumption):ab,ti 0R (VO_2max_):ab,ti 0R (hemoglobin):ab,ti 0R (haemoglobin):ab,ti 0R (Hb):ab,ti

#3 (randomized controlled trial):ab,ti,kw OR (randomized):ab,ti,kw OR (placebo):ab,ti,kw

#4 #1 and #2 and #3

Note: We searched 752 articles while using RefMan-(RIS) as the filter.

**Web of Science**

#1 TS=(intermittent hypoxic or interval training or intermittent hypoxia)

#2 TS=(aerobic capacity or aerobic endurance or aerobic stamina or cardio or maximal oxygen consumption or VO_2max_ or hemoglobin or haemoglobin or Hb)

#3 TS=(randomized controlled trial or randomized or placebo)

#4 #1 AND #2 AND #3

Note: We searched 1331 articles while using Web of science as the filter.
